# Short-term CFTR inhibition reduces islet area in C57BL/6 mice

**DOI:** 10.1038/s41598-019-47745-w

**Published:** 2019-08-02

**Authors:** Dawood Khan, Ryan Kelsey, Rashmi R. Maheshwari, Virginia M. Stone, Annie Hasib, Fiona N. Manderson Koivula, Aoife Watson, Stephen Harkin, Nigel Irwin, James A. Shaw, Neville H. McClenaghan, Viktória Venglovecz, Attila Ébert, Malin Flodström-Tullberg, Michael G. White, Catriona Kelly

**Affiliations:** 1 Northern Ireland Centre for Stratified Medicine, School of Biomedical Sciences, Ulster University, C-TRIC Building, Altnagelvin Hospital Campus, Glenshane Road, Derry/Londonderry, Northern Ireland UK; 2 Institute of Cellular Medicine, Newcastle University, Framlington Place, Tyne and Wear UK; 3 The Center for Infectious Medicine, Department of Medicine Huddinge, Karolinska Institutet, Karolinska University Hospital Huddinge, Stockholm, Sweden; 4 School of Biomedical Sciences, University of Ulster, Cromore Road, Coleraine, Northern Ireland UK; 5 Department of Pharmacology and Pharmacotherapy, University of Szeged, Szeged, Hungary

**Keywords:** Cell biology, Diabetes complications

## Abstract

Cystic fibrosis-related diabetes (CFRD) worsens CF lung disease leading to early mortality. Loss of beta cell area, even without overt diabetes or pancreatitis is consistently observed. We investigated whether short-term CFTR inhibition was sufficient to impact islet morphology and function in otherwise healthy mice. CFTR was inhibited in C57BL/6 mice via 8-day intraperitoneal injection of CFTRinh172. Animals had a 7-day washout period before measures of hormone concentration or islet function were performed. Short-term CFTR inhibition increased blood glucose concentrations over the course of the study. However, glucose tolerance remained normal without insulin resistance. CFTR inhibition caused marked reductions in islet size and in beta cell and non-beta cell area within the islet, which resulted from loss of islet cell size rather than islet cell number. Significant reductions in plasma insulin concentrations and pancreatic insulin content were also observed in CFTR-inhibited animals. Temporary CFTR inhibition had little long-term impact on glucose-stimulated, or GLP-1 potentiated insulin secretion. CFTR inhibition has a rapid impact on islet area and insulin concentrations. However, islet cell number is maintained and insulin secretion is unaffected suggesting that early administration of therapies aimed at sustaining beta cell mass may be useful in slowing the onset of CFRD.

## Introduction

Cystic fibrosis (CF) is an autosomal recessive condition caused by mutations in the CF transmembrane conductance regulator (CFTR) gene, which encodes a cAMP activated chloride channel of the same name. CFTR is an apical membrane Cl^−^/HCO_3_^−^ channel, which controls epithelial cell salt and fluid secretion across the plasma membrane. Abnormal CFTR leads to dehydrated, acidic secretions, which drive CF pathogenesis^1^. The vast majority of morbidity and mortality in CF results from lung disease where a build up of viscous mucous leads to bacterial colonisation of the airways and a decline in lung health. However, CFTR is highly expressed in various organs including the airways, pancreas and intestine^2^.

CF-related diabetes (CFRD) is the largest extra-pulmonary co-morbidity in the CF population and significantly accelerates lung decline. In comparison to normoglycaemic individuals, patients with CFRD have worse pulmonary function, more frequent and severe pulmonary exacerbations, and a greater prevalence of bacteria in the sputum^3^. CFRD is present in 30–50% of adults with CF, and prevalence is rapidly increasing as CF life expectancy improves^4^. The pathogenesis of CFRD remains poorly understood and extensive debate exists as to the role of CFTR in islet function and insulin secretion. Several investigators report CFTR channel activity in mouse^5–8^ and human islets^5,7^ and show insulin and glucagon secretory defects in the absence of functional CFTR^5–9^. Conversely, others report that CFTR expression is very low or absent in human pancreatic endocrine cells^10–12^ and suggest that impairments in insulin secretion likely result from decreased islet mass, intra-islet inflammation or exocrine-derived inflammatory mediators^10,11^.

Notwithstanding, the debate surrounding the location of CFTR within the pancreas, compelling evidence from human tissue and CF animal models consistently reports reductions in beta cell area and insulin content. Seminal studies in the pancreas of CF patients showed marked reductions in beta cell area irrespective of whether diabetes was present or not^13,14^. This was confirmed in the CF ferret where altered islet morphology and function were reported at birth. In this model, a significant increase in the number of small islets was observed in neonates^15^. Subsequent studies have repeatedly shown reductions in islet mass or beta cell area in animal models of CF^16–19^ and in post mortem pancreas from patients with CF^10,20^ including children under the age of 4 years^20^. In the latter study, beta cell area was reduced by as much as 50% in children less than 4 years old when compared with age-matched controls and this was shown to be independent of the degree of exocrine pancreatic fibrosis or the presence/absence of diabetes^20^. It is therefore clear that decreased islet and beta cell area occurs early in the pathogenesis of CF. In this study we wished to explore just how quickly this happened through the creation of a mouse model of short-term CFTR inhibition. To determine if any observed impact of CFTR was dependent on, or exacerbated by, hyperglycaemia, mice were exposed to CFTRinh172 alone or in combination with streptozotocin. Streptozotocin-induced hyperglycaemia occurs via beta cell toxicity leading to a near absolute insulin requirement. The inhibition of CFTR in otherwise healthy animals allows for the assessment of whether CFTR has an essential role to play in the maintenance of islet area, whether loss of islet area could be attributed to developmental deficiencies, or whether loss of islet area is solely driven by a hyperinflammatory state in the pancreas.

## Results

### Short-term CFTR inhibition impacts weight gain

**Figure 1:**
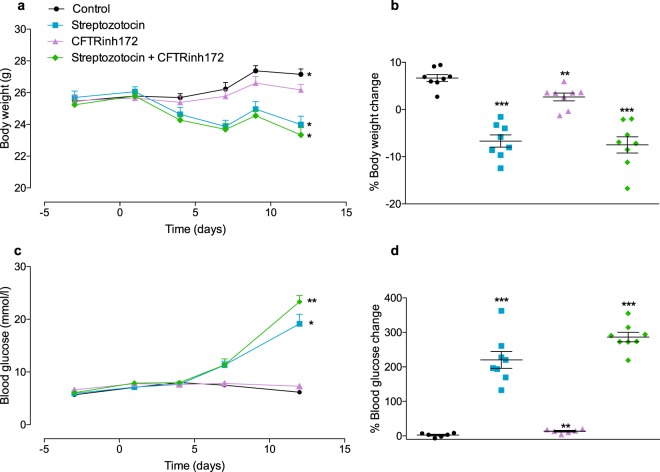
Body weight and blood glucose concentrations. Mice were treated with vehicle control, streptozotocin, CFTRinh172, or a combination of the two and (**a**) body weight over the duration of the study (Days -3, 1, 4, 7, 9 and 12), (**b**) percentage change in body weight from the beginning to the end of the study (Day -3 to Day 12), (**c**) blood glucose concentrations over the duration of the study (Days -3, 1, 4, 7 and 12), and (**d**) percentage change in blood glucose concentrations from the beginning to the end of the study (Day -3 to Day 12) were measured. Data are presented as mean ± SEM (n = 7–8 mice for all experiments). (**a**,**c**) *p < 0.05 compared with values at Day -3 (*t* test). (**b**,**d**) **p < 0.01, ***p < 0.001 compared to control animals (One-way ANOVA).

Body weight change over the duration of the study is shown in Fig. 1a and the percentage change in body weight from Day -3 to Day 12 is shown in Fig. 1b. Control animals had significant weight gain over the course of the study (*p* < 0.05, Fig. 1a). Animals treated with CFTRinh172 alone showed a non-significant trend to weight gain (Fig. 1a). However, their percentage body weight gain from the start to the end of the study was significantly lower (60.3 ± 31.17% reduction; *p* < 0.01) than that of control animals (Fig. 1b). Streptozotocin treatment was associated with significant and comparable weight loss in the presence or absence of CFTRinh172 co-administration (Fig. 1a,b). Cumulative food and water intake were lower than in control animals during administration of STZ and CFTRinh172 alone or a combination (Fig. S2, ESM). Treatment of animals with STZ, CFTRinh172 or a combination of the two did not impact bone mineral content, bone mineral density or lean mass (Fig. S2, ESM). However, STZ treatment, alone or in combination with CFTRinh172 was associated with a reduction (*p* < 0.01) in fat mass (Fig. S2, ESM).

### Short-term CFTR inhibition does not induce hyperglycaemia or glucose intolerance

Control animals showed no change in blood glucose concentration over the course of the study. Animals treated with CFTRinh172 alone were not hyperglycaemic (Fig. 1c) but displayed a modest increase in blood glucose over the duration of the study (13.75 ± 7.28% increase between Day-3 and Day 12, *p* < 0.01, Fig. 1d). STZ treatment was associated with significant hyperglycaemia (Fig. 1c). STZ and CFTRinh172 co-administration led to a greater increase in glucose in comparison to STZ alone (Fig. 1d).

**Figure 2:**
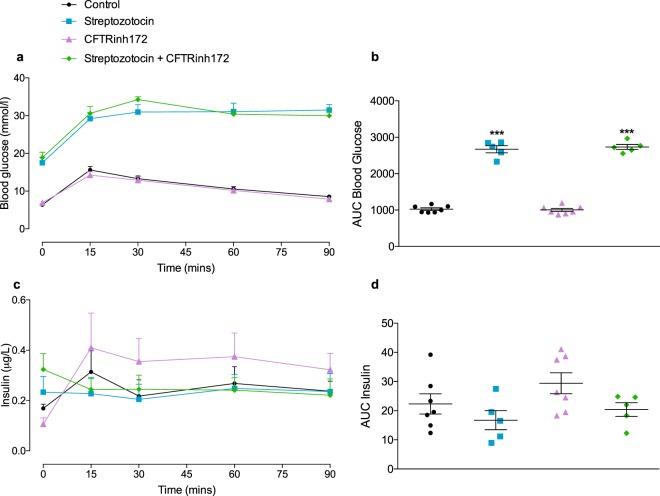
Glucose tolerance. Mice were treated with vehicle control, streptozotocin, CFTRinh172, or a combination of the two. Intraperitoneal glucose tolerance tests were undertaken on study Day 12 (**a**) Blood glucose concentrations and (**c**) plasma insulin concentrations were assessed immediately before and 15, 30, 60 and 90 minutes after intraperitoneal administration of glucose (18 mmol/kg body weight). Respective areas under the curve for (**b**) blood glucose and (**d**) plasma insulin AUCs are also shown. Data are presented as mean ± SEM (n = 5–7 mice for all experiments). ***p < 0.001 compared to control animals (One-way ANOVA).

Glucose tolerance was tested over 90 minutes following i.p. administration of a glucose load on Day 12 of the study. Administration of CFTRinh172 alone had no significant impact on glucose tolerance (Fig. 2a,b). Multiple low dose streptozotocin alone, or in combination with CFTRinh172, was associated with fasting hyperglycaemia and abnormal glucose tolerance test (Fig. 2a,b). There were no significant differences between groups in fasting (Fig. 2c) or area under the curve serum (AUC) insulin (Fig. 2d). Consistently, calculation of HOMA-IR (Fig. S3a, ESM), HOMA-B (Fig. S3b, ESM), QUICKI (Fig. S3c, ESM) and measurement of phosphorylated IRS-1 in liver lysates (Fig. S3d), showed that animals treated with STZ, either alone or in combination with CFTRinh172, displayed evidence of insulin resistance and poor insulin sensitivity. Mice treated with CFTRinh172 alone were insulin sensitive (Fig. S3, ESM).

### Short-term CFTR inhibition reduces peripheral and pancreatic insulin concentrations

**Figure 3:**
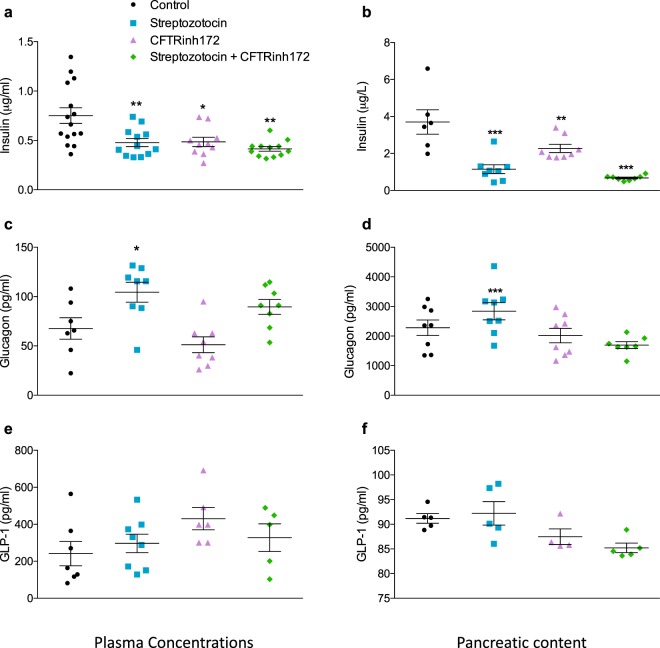
Islet hormone concentrations. Peripheral plasma concentrations of (**a**) insulin (n = 10–15), (**c**) glucagon (n = 6–8), and (**e**) GLP-1 (n = 5–8) were assessed on Day 12 of the study. Additionally, the pancreatic content of (**b**) insulin (n = 6–8), (**d**) glucagon (n = 7–8), and (**f**) GLP-1 (n = 5) were assessed in protein extracted from pancreatic tissue. All observations were made in mice fasted for 2 hours. Data are presented as mean ± SEM for a given number of mice (n) as indicated above. *p < 0.05, **p < 0.01 and ***p < 0.001 compared to control animals (One-way ANOVA).

Plasma insulin, glucagon, and GLP-1 concentrations were assessed on the final day of the study following 2 hours fasting. In addition, the pancreatic content of each islet hormone was also investigated. Insulin concentrations (Fig. 3a,b) were reduced (*p* < 0.01) and glucagon concentrations (Fig. 3c,d) were elevated (*p* < 0.05) in STZ-treated animals. The current data show that short-term CFTR inhibition resulted in significant reductions in plasma insulin concentrations (36.40 ± 9.62% reduction, *p* < 0.01, Fig. 3a) and pancreatic insulin content (39.42 ± 9.74% reduction, *p* < 0.01, Fig. 3b). CFTR inhibition had little impact on glucagon or GLP-1 concentrations (Fig. 3).

### Short-term CFTR inhibition reduces islet cell size, not number

**Figure 4:**
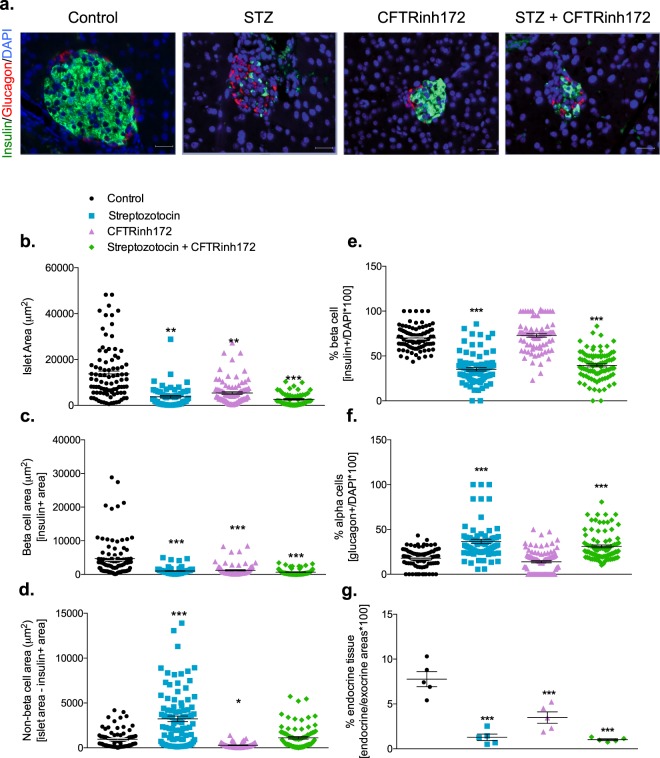
Islet size and composition. (**a**) Representative images of pancreatic islets from mice treated with vehicle control, streptozotocin, CFTRinh172, or a combination of the two and stained for insulin (green), glucagon (red), and the nuclear stain DAPI (blue). (**b**) Islet area (µm^2^), (**c**) beta cell area [insulin positive area] (µm^2^), (**d**) non-beta cell area [non-insulin positive area] (µm^2^), (**e**) beta cell number as a percentage of total islet cells [insulin positive cells/DAPI count] and (**f**) alpha cell number as a percentage of total islet cells [glucagon positive cells/DAPI count]. Data are presented as mean ± SEM (n = 5 mice) and calculated from two sections of tissue per animal taken at least 200 µm apart. A minimum of 100 islets were counted from a total of 5 animals (at least 20 islets per animal). Each data point on the graphs represents one islet. **p < 0.01 and ***p < 0.001 com*p*ared to controls animals (One-way ANOVA).

Sectioned pancreata were stained for insulin and glucagon (Fig. 4a). Islet area, beta cell area (insulin positive area) and non-beta cell area (total islet area minus insulin positive area) were calculated. Animals treated with STZ showed a reduction in islet size (*p* < 0.01, Fig. 4b), accompanied by a reduction in beta cell area (*p* < 0.001, Fig. 4c) and a significant increase in non-beta cell area (*p* < 0.001, Fig. 4d). Short-term CFTR inhibition caused significant reductions in islet area (69 ± 20% reduction, *p* < 0.001, Fig. 4b), beta cell area (72 ± 20% reduction, *P* < 0.001, Fig. 4c) and non-beta cell area (56 ± 23% reduction, *p* < 0.001, Fig. 4d). To address whether the reductions in islet size resulted from changes in the number of islet cells or changes in the size of islet cells, insulin or glucagon positive cells were counted and expressed as a percentage of total islet cells (total DAPI counts per islet). As shown in Fig. 4e,f, the proportion of insulin and glucagon-positive cells per islet did not differ between control animals and those treated with CFTRinh172 alone. In contrast, the proportion of beta cells per islet decreased and the proportion of alpha cells increased in STZ-treated animals with and without CFTRinh172 co-administration. Finally, we assessed the percentage endocrine area (calculated as [endocrine area/exocrine area*100] where endocrine area comprised the cumulative area for all islets present in each section of tissue) for all animals (Fig. 4g). The mean endocrine area for control animals was 7.7 ± 0.8%. The percentage endocrine area of all other groups was significantly (*p* < 0.001) lower than that of control animals. The endocrine area of CFTRinh172-treated animals was 3.4 ± 0.6% (Fig. 4g).

### Short-term CFTR inhibition does not alter beta-cell transcription factor expression

**Figure 5:**
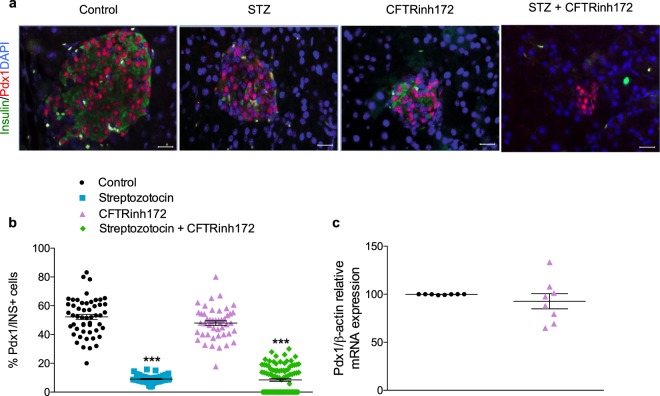
Pdx-1 expression. (**a**) Representative images of pancreatic islets from mice treated with vehicle control, streptozotocin, CFTRinh172, or a combination of the two and stained for insulin (green), Pdx-1 (red), and the nuclear stain DAPI (blue). (**b**) Number of Pdx-1 positive cells as a percentage of beta cells (insulin positive cells). Data are presented as mean ± SEM (n = 5 mice). A minimum of 50 islets were counted from a total of 5 animals (at least 10 islets per animal). Each data point on the graphs represents one islet. ***p < 0.001 compared to control animals (One-way ANOVA). (**c**) Relative *PDX1* mRNA expression in the isolated islets of control animals and those treated with CFTRinh172 alone. Data are presented as mean ± SEM (n = 8).

**Figure 6:**
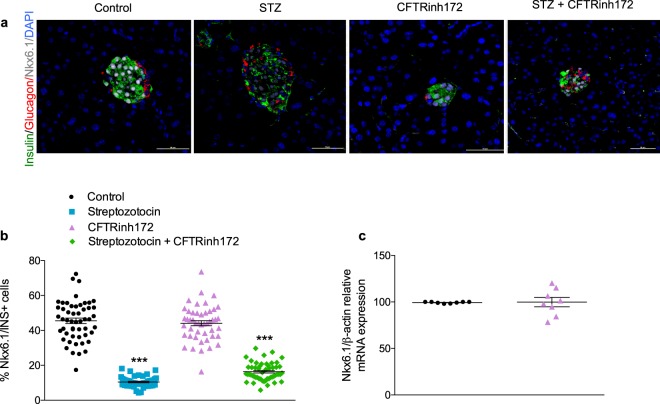
Nkx6.1 expression. (**a**) Representative images of pancreatic islets from mice treated with vehicle control, streptozotocin, CFTRinh172, or a combination of the two and stained for insulin (green), glucagon (red), Nkx6.1 (grey), and the nuclear stain DAPI (blue). (**b**) Number of Nkx6.1 positive cells as a percentage of beta cells (insulin positive cells). Data are presented as mean ± SEM (n = 5 mice). A minimum of 50 islets were counted from a total of 5 animals (at least 10 islets per animal). Each data point on the graphs represents one islet. ***p < 0.001 compared to control animals (One-way ANOVA). (**c**) Relative *NKX6-1* mRNA expression in the isolated islets of control and CFTR-inhibited mice. Data are presented as mean ± SEM (n = 8).

The proportion of beta cells expressing the transcription factors Pdx-1 and Nkx6.1 was determined by expressing the number of cells co-stained for insulin and either Pdx-1 or Nkx6.1 as a percentage of total insulin positive cells. Multiple low-dose STZ, alone or in combination with CFTRinh172 significantly (*p* < 0.01 to *p* < 0.001) reduced the percentage of Pdx-1 (Fig. 5a,b) and Nkx6.1 (Fig. 6a,b) positive beta cells in C57BL/6 mice. No change in the percentage of Pdx-1 (Fig. 5a,b) or Nkx6.1 (Fig. 6a,b) positive beta cells was observed in animals treated with CFTRinh172 alone. Consistently, the mRNA expression of *PDX1* (Fig. 5c) or *NKX6-1* (Fig. 6c) in isolated islets did not differ between control and CFTR-inhibited animals.

### Insulin secretion is not influenced by short-term CFTR inhibition

**Figure 7:**
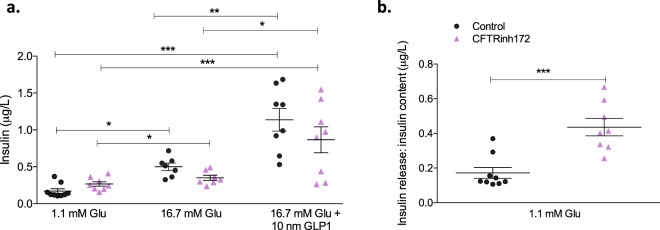
Glucose stimulated insulin secretion. Islets were isolated from animals treated with vehicle control or CFTRinh172 and exposed to 1.1 mM glucose, or 16.7 mM glucose ± 10 nM GLP-1 for 1 h. (**a**) Static insulin release was measured by ELISA with *p < 0.05, **p < 0.01 and ***p < 0.001 compared to either 1.1 mM or 16.7 mM glucose as indicated in the Figure (one-way ANOVA). (**b**) Data was adjusted for pancreatic insulin content under basal (1.1 mM) glucose conditions with ***p < 0.001 compared to corresponding control animals (*t* test). Data are presented as mean ± SEM (n = 8–10 for all experiments).

Islets were isolated from mice treated with CFTRinh172 alone or vehicle control and challenged with glucose or the cAMP agonist GLP-1. In all instances, elevation of glucose concentration in the presence or absence of GLP-1 evoked significant increases in insulin release compared with the corresponding control under basal conditions (Fig. 7a). No significant differences were observed in glucose-stimulated insulin secretion or GLP-1 potentiated insulin secretion between vehicle controls and animals treated with CFTRinh172 when absolute insulin concentrations were measured (Fig. 7a). Additionally, no significant differences were detected in the mRNA expression of several glucose sensing genes (*SCLC2A2, GCK, ABCC8, KCNJ11*) in islets from CFTR-inhibited mice (Fig. S4, ESM). However, when insulin secretion was adjusted for pancreatic insulin content, the fraction of insulin released from islets isolated from CFTRinh172-treated mice was significantly higher (*p* < 0.001) than that of vehicle controls under basal (1.1 mM) glucose concentrations (Fig. 7b).

### Short-term CFTR inhibition generates pancreatic and intestinal phenotypes that are consistent with CF disease

**Figure 8:**
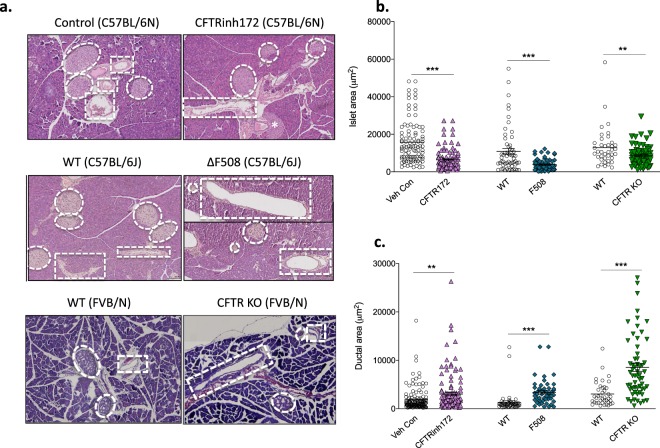
Pancreatic morphology. Representative H&E stained sections of the (**a**) pancreas of CFTRinh172-treated mice and vehicle controls (both n = 5), transgenic ΔF508 mice and wild-type controls (both n = 3), CFTR KO mice (n = 3) and wild-type controls (n = 2). Islets are denoted by dashed white circles with pancreatic ducts identified by dashed rectangles. (**b**) Islet area and (**c**) pancreatic ductal area have been quantified. Values represent mean ± SEM calculated from two sections of tissue per animal taken at least 200 µm apart. A minimum of 20 data points (at least 10 per section) were collected for each animal. Each data point on the graphs represents one islet/duct. **p < 0.01 and ***p < 0.001 com*p*ared to corresponding control/wild type animals (*t* test). Please note that the representative image of the pancreas shown for the ΔF508 mice is a composite. The complete image is presented in Fig. S6, ESM.

H&E staining of the pancreas revealed a significant (*p* < 0.001) reduction in islet size (Fig. 8a,b) and an increase (*p* < 0.01) in ductal area (Fig. 8a,c) in CFTR-inhibited animals consistent with observations from ΔF508 and CFTR KO mice (Fig. 8). Pancreatic inflammation is consistently reported in CF disease and recent work has shown that IL-6 plays a key role in islet dysfunction in CF ferrets^11^. In the current study, short-term CFTR inhibition did not cause an increase in IL-6 concentrations in the pancreas (Fig. S5, ESM) and there was little histological evidence of inflammatory cell infiltrate (See Fig. S7, ESM). Finally, intestinal villus height was not influenced by short-term CFTR inhibition, consistent with the ΔF508 model (Fig. S8, ESM).

Sirius red/fast green staining was undertaken to determine if there was any evidence of early stage fibrosis in the tissues of animals treated with CFTRinh172. Despite occasional evidence of additional collagen deposition around the pancreatic ducts and within the basement membrane of the intestine, the overall % area stained for Sirius red did not differ between control and CFTR-inhibited animals (Fig. S9, ESM).

## Discussion

In the present study we generated a mouse model of short-term (8 day) CFTR inhibition using low-dose treatment with the pharmacological CFTR inhibitor, CFTRinh172, in otherwise healthy mice. CFTRinh172 is a small molecule containing 2-thioxo-4-thiazolidinone core that acts to inhibit CFTR Cl^−^ channel function by binding to the first nucleotide-binding domain of CFTR and stabilising closed channel state^21,22^. Following short-term administration of this inhibitor, we observed marked and consistent reductions in islet size that were associated with reduced pancreatic insulin content and reduced circulating insulin concentrations. Our data show that loss of CFTR function, even on a short-term basis is sufficient to mediate reductions in islet size and insulin content.

Previous studies have suggested that beta cell dysfunction and insulin deficiency results in impaired glucose tolerance in the majority of CF patients^23^. Glucose abnormalities are frequently observed in children with CF and in very rare cases, have been reported in infants under the age of 2^24–27^. In the current study, CFTR-inhibited animals displayed a modest increase in blood glucose concentrations but were not diabetic. The animals also had normal glucose tolerance in response to an intraperitoneal glucose challenge. None of the observed effects of CFTR inhibition were dependent on, or exacerbated by, hyperglycaemia.

Islet area was reduced by some 70% in the present study, with beta cell and non-beta cell areas similarly reduced. Recently, Rotti *et al*.^18^ detailed changes in islet composition in CF ferrets of increasing age and phases of glycaemic regulation. In the ferret model, newborn animals and those up to the age of 4 months, displayed relatively similar distribution of insulin, glucagon and somatostatin positive cells. When the number of insulin, or glucagon-positive cells were counted as a percentage of total islet cells in this model of short-term CFTR inhibition, it was evident that the reduction in islet size resulted from smaller islet cells rather than loss of cells. This is consistent with recent observations in the ΔF508 mouse^19^ and raises the attractive possibility that early interventions to prevent reductions in islet mass may assist in the maintenance of normal islet size and function in CF. In line with the reductions in islet size, insulin content and peripheral insulin concentrations were also significantly reduced. Glucagon and GLP-1 concentrations were spared in this model.

Reductions in insulin secretion in the face of glucose challenge have been reported in several CF animal^15,16,19,28^ and cell models^5–9^, and in patients with CF^28–33^. Fontes *et al*.^17^ report that impairments in insulin secretion from the isolated islets of ΔF508 mice result from reductions in insulin content associated with loss of beta cell mass. Short-term CFTR inhibition had little impact on glucose-stimulated, or GLP-1 potentiated, insulin secretion in the current study even in the absence of any adjustment for insulin content. However, it should be noted that the inhibitor used here is reversible and therefore the washout period of 7 days may mean that an acute secretory defect was missed. However, it is noteworthy that the reductions in islet size and insulin content did not impact insulin secretion in response to challenge. Importantly, the fraction of insulin secreted under basal conditions was higher in CFTR-inhibited mice than vehicle controls, consistent with recent observations in the CF ferret^11^. This may explain the ability of these islets to function well in the face of GLP-1 challenge despite significant reductions in size and insulin content.

Prior use of CFTRinh172 *in vivo* caused defects in the nasal epithelial ion transport in mice^34^, and reduced efficacy in submucosal gland fluid secretion in pig and human airways^35^. Nonetheless, we were conscious of the use of a pharmacological inhibitor of CFTR, which has the potential to generate off-target effects^11,36,37^. We have taken several steps to minimise this risk including low-dose use of the inhibitor, which has been reported to limit the potential for aberrant effects^36^. The literature suggests that mice will tolerate up to 50 mg/kg per day for 1 week, or 3 mg/kg CFTRinh172 twice daily for a period of 6 weeks with little evidence of toxicity^38^. In the current study, we used the lower dose and administered the treatment once daily. Animals were also allowed a wash-out period of one week before most of the data presented here (including islet hormone concentrations, insulin secretion and morphological analyses) were assessed. Although CFTR-inhibited animals weighed a little less than the control group, they otherwise appeared to tolerate the treatment well.

To further validate our model of short-term CFTR inhibition, we sought to characterise the phenotype created following CFTR inhibition and to compare this directly with a transgenic CF mouse model (ΔF508)^39^, a CFTR KO model^40^ and the known literature on CF disease. The pancreatic and intestinal phenotypes of CFTR-inhibited mice were largely consistent with CF disease. Islet size was reduced in CFTR-inhibited animals, and the ΔF508 and CFTR KO animals, consistent with the literature^17,19^. In addition, ductal dilatation, a hallmark of CF disease, was evident in all three mouse models studied here. Intestinal villi height is occasionally reduced in CF^41^. However, in both the CFTR-inhibited model and the ΔF508 model, we observed no change in villi height.

Recent work suggested that pancreatic IL-6 concentrations contribute to islet dysfunction in CF ferrets^11^. However, IL-6 concentrations were not elevated in our model of CFTR inhibition and there was little histological evidence of inflammatory cell infiltrate within the islets. The absence of inflammation in this model does not negate the role of pancreatitis and intra-islet inflammation in the pathogenesis of CFRD. Indeed, it seems implausible that the pancreatic environment in advanced CF disease would not impact islet function. However, these findings show that islets are impacted early after loss of CFTR, and suggest that these changes, even in the absence of overt inflammation/proinflammatory cytokine signalling, may contribute to islet dysfunction.

Here we report that short-term CFTR inhibition does not cause clinically significant glucose abnormalities, nor is it associated with spontaneous development of diabetes, a long-term defect in the secretory capacity of islets, pancreatic inflammation or fibrosis. This is in contrast with much of the published literature discussed above. It is likely that the acute duration of CFTR inhibition examined in this study was insufficient to trigger several defects often associated with age. For example, Fontes *et al*.^17^ report glucose intolerance in 24-week old ΔF508 mice, but not in 12-week old animals. Moreover, we found little evidence of pancreatic inflammation in CFTR-inhibited animals as has been reported in transgenic animals^11^ and in adult CF patients^10,20^ where chronic disease has persisted for periods far beyond the acute 8-day duration of CFTR inhibition examined here. Consistently, pancreatic inflammation is less commonly observed in CF children^20^. Furthermore, the washout period following CFTR inhibition and the reversible nature of the inhibitor itself may mean that an acute functional secretory defect has been missed.

In conclusion, the present observations provide further evidence that CFTR is essential to the maintenance of islet size and insulin content within the pancreas. The current model of global CFTR inhibition does not allow for distinction between islet intrinsic versus extrinsic roles of CFTR. However, the data shows that inhibition of CFTR has a rapid impact on islet size. Importantly, the use of healthy adult mice in this study excludes the possibility that early loss of islet mass in the absence of functional CFTR is purely a developmental issue. The absence of inflammation in our model raises the possibility that additional trophic factors beyond the pancreas may be regulating islet mass in this state. The observations that (1) islet cell number is maintained even in the face of a reduction in area and that (2) the secretory capacity of islets is sustained despite significant reductions in insulin content, suggest that early administration of therapies aimed at sustaining beta cell mass may be useful in slowing the onset of CFRD.

## Methods

### Animal models

Animal studies were conducted in male C57BL/6 mice exposed to a pharmacological inhibitor of CFTR, CFTRinh172. Experiments were licensed according to UK Home Office regulations (UK Animals Scientific Procedures Act 1986) and associated guidelines (EU Directive 2010/63/EU) and approved by the University of Ulster Animal Ethics Review Committee. The pancreatic (and where possible, intestinal) phenotypes of CFTR-inhibited mice were compared with those of Cftr^tm1EUR^ mice^39^, hereafter referred to as ΔF508 mice, and CFTR KO mice^40^. Further information on the age, genetic background, and the husbandry and maintenance of the mice is provided in the methods section of the electronic supplementary material (ESM).

### *In vivo* studies

C57BL/6 mice were age-matched and grouped based on their fasted blood glucose and body weight. The dosing regimen is described in Fig. S1 (ESM). In brief, animals received once daily intraperitoneal (i.p.) injections of vehicle control (DMSO) or CFTRinh172 (3 mg/kg body weight) for 8 consecutive days starting on Day -3. To determine if the effects of CFTR inhibition were dependent on, or exacerbated by, hyperglycaemia, CFTRinh172 was administered alone or in combination with a low dose streptozotocin (STZ; 50 mg/kg body weight) regimen, which began on Day 0 of the study and continued for 5 consecutive days. This resulted in the creation of 4 groups of animals: (1) vehicle control; (2) STZ-treated; (3) CFTRinh172-treated; and (4) STZ + CFTRinh172-treated. Specific information on the routine measurement of body mass composition, blood glucose and glucose tolerance tests are given in Fig. S1 and in the methods section of the ESM.

After a 7-day washout period, mice were killed by Schedule 1 methods and pancreata, intestines and terminal blood samples collected. Pancreata were halved longitudinally from head to tail with one half used for histology and the other half used for islet isolation or determination of islet hormone content as described below and in the ESM.

### Determination of islet hormone concentrations

Commercially available ELISAs were used to measure insulin (Ultra-sensitive murine insulin ELISA, Mercodia), glucagon (Glucagon Quantikine ELISA Kit, R&D Systems) and GLP-1 (GLP-1 EIA Kit, Sigma-Aldrich) concentrations according to the manufacturers’ instructions. Further details on the processing of plasma and pancreatic protein extract for assessment of islet hormone concentrations is given in the methods section of the ESM.

### Islet isolation and determination of insulin secretion

Islets were isolated from vehicle control and CFTR-inhibited mice by collagenase digestion, as previously described^42^. Islets were cultured overnight in RMPI supplemented with 10% FBS and 1% Penicillin/Streptomycin at 37 °C and 5% CO_2_ prior to experimentation. Batches of ten islets underwent static incubation in basal glucose (1.1 mM D-glucose) for 1 hour, followed by exposure to test solution (1.1 mM D-glucose, or 16.7 mm D-glucose ± 10 nM GLP-1) for a further 1 hour. Supernatants were collected and the secretion of insulin assessed by ELISA (Ultra-sensitive murine insulin ELISA, Mercodia).

### Expression of islet regulatory genes

The mRNA expression of islet regulatory genes was assessed by qPCR. mRNA was extracted from isolated mouse islets^42^ using a RNeasy Mini Kit following manufacturer’s instructions (Qiagen, UK). mRNA (200 ng) was reverse transcribed to cDNA using transcriptor first strand cDNA synthesis kit (Roche, Burgess Hill, UK). UK). qPCR was performed on a Lightcycler 480 System (Roche, UK) using custom designed probes (Roche). Probe IDs are provided in Table S1 (ESM) and further information on cycling conditions and quantification of relative expression are available in the methods section of the ESM.

### Immunofluorescent staining of pancreatic tissue

Pancreatic tissues were immediately fixed, processed and embedded in paraffin wax using an automated tissue processor (Leica TP1020, Leica Microsystems, Nussloch, Germany), as described previously^43^. Tissues were stained with appropriate primary (Table S2, ESM) and secondary antibodies (Table S3, ESM). Full details on tissue processing and staining are given in the ESM. Slides were mounted with anti-fade mounting medium (Vectashield, Vector Laboratories) with DAPI and viewed using a Nikon A1 confocal microscope (Nikon UK Limited, Surry, United Kingdom). All staining procedures and image analysis were carried out in a blinded manner.

### Phenotypic characterisation of pancreatic and intestinal tissue

Pancreata and intestines from mice treated with CFTRinh172, ΔF508, and CFTR KO mice and corresponding control animals were fixed, processed and sectioned as described. Haematoxylin and Eosin staining (H&E) was conducted as previously described^44^. Quantification of collagen deposition was conducted using the sirius red/fast green method. Briefly, 4 μm sections were deparaffinised, rehydrated and incubated in 0.1% Fast Green for 5 minutes. Following rinsing in distilled water, sections were incubated in 0.5% Sirius Red for 10 minutes before counterstaining with Haematoxylin, rinsed in Scott’s Tap Water and mounted with Pertex (CellPath).

### Image analysis

For measurements of islet size, beta cell area, non-beta cell area and endocrine:exocrine area, two sections of tissue separated by at least 200 µm were analysed for each animal. Measurements from a minimum of 20 data points (i.e. at least 10 islets per section) were collected per animal from at least 5 different mice. Nikon NIS-AR image analysis software (NIS Elements) was used to analyse islet parameters including islet area, alpha cell area (glucagon positive area) and beta cell area (insulin positive area), expressed as µm^2^. Islet parameters were determined using the ‘closed polygon’ tool in analysis software. The number of cells per islet were manually counted using cell counter in ImageJ software. For assessment of the percentage Pdx-1 and Nkx6.1 positive cells within the islet, analyses were performed on at least 10 islets per animal from five different mice and further validated by qPCR.

### Statistical analysis

Statistical analysis was performed using GraphPad PRISM (La Jolla, CA, USA; version 7). Data are presented as mean ± SEM for a given number of observations (n) as indicated in the Figures. All samples were numbered and blinded, and researchers were unblinded only when analyses were complete. Differences between groups were compared using one-way ANOVA or unpaired 2-tailed Student’s *t* test as appropriate. Statistical significance was accepted at *p* < 0.05.

## Supplementary information

Electronic Supplementary Material

## Data Availability

The datasets generated during and/or analysed during the current study are available from the corresponding author on reasonable request.
