# Supplementary material for: Short-term CFTR inhibition reduces islet area in C57BL/6 mice
Source: Sci Rep. 2019 Aug 2;9:11244. doi: 10.1038/s41598-019-47745-w (PMC6677757; doi:10.1038/s41598-019-47745-w)
Supplement: Supplementary file 1 — Electronic Supplementary Material [file 41598_2019_47745_MOESM1_ESM.pdf]

ELECTRONIC SUPPLEMENTARY MATERIAL

**Short-term CFTR inhibition reduces islet area in C57BL/6 mice.**

Dawood Khan,[1] Ryan Kelsey,[1] Rashmi R. Maheshwari,[2] Virginia M. Stone,[3] Annie Hasib,[4] Fiona N. Manderson Koivula,[1] Aoife Watson,[1] Stephen Harkin,[1] Nigel Irwin,[4] James A. Shaw,[2] Neville H. McClenaghan,[4] Viktória Venglovecz,[5] Attila Ébert,[5] Malin Flodström-Tullberg,[3] Michael G. White,[2] Catriona Kelly.[1]

1. Northern Ireland Centre for Stratified Medicine, School of Biomedical Sciences, Ulster Unviersity, C-TRIC Building, Altnagelvin Hospital Campus, Glenshane Road, Derry/Londonderry, Northern Ireland, UK
2. Institute of Cellular Medicine, Newcastle University, Framlington Place, Tyne and Wear, UK
3. The Center for Infectious Medicine, Department of Medicine Huddinge, Karolinska Institutet, Karolinska University Hospital Huddinge, Stockholm, Sweden.
4. School of Biomedical Sciences, Ulster University, Cromore Road, Coleraine, Northern Ireland, UK
5. Department of Pharmacology and Pharmacotherapy, University of Szeged, Szeged, Hungary

*Corresponding author: Catriona Kelly, Northern Ireland Centre for Stratified Medicine, School of Biomedical Sciences, Ulster University, C-TRIC Building, Altnagelvin Hospital Campus, Glenshane Road, Derry/Londonderry, Northern Ireland, UK. E-mail: c.kelly@ulster.ac.uk Tel: +44(0)28 7167 5674.

## METHODS

### Animal models

*Short-term CFTR inhibition*

Animal studies were conducted in male C57BL/6NHsd (10-12 weeks old, Envigo Ltd, UK) maintained on a standard rodent diet (10% fat, 30% protein and 60% carbohydrate, Trouw Nutrition, UK). All animals were housed individually in an air-conditioned room at 22 ± 2 °C with a 12 hour light and dark cycle and had access to food and water *ad lib*. All *in vivo* studies on C57BL/6 mice were conducted as described in the main text and were carried out in accordance with the UK Animals (Scientific Procedures) Act 1986.

*ΔF508 mouse model*

CFTR$^{tm1EUR}$ mice,[1] hereafter referred to as ΔF508 mice, are on a C56BL/6J background. The ΔF508 mice were a kind gift from Dr Bob Scholte, Erasmus MC, Rotterdam and were embryo derived upon arrival at the Karolinska University Hospital Huddinge animal facility, Sweden. Male and female ΔF508 mice (aged 8-12 weeks) were housed in specific pathogen-free conditions in isolator cages with between 2-5 mice per cage. ΔF508 mice were bred as heterozygotes and genotyping was performed as described.[1] Most of the mice were untreated however a few were used as controls in a separate experiment and received 200μl PBS buffer by i.p injection two or four hours prior to euthanasia.[2] Mice were anaesthetised with isofluorane and then killed by cervical dislocation. The mice were housed, and experiments were performed according to both local and national regulations and ethical permission for the studies was granted by the local ethics committee.

*CFTR KO mouse model*

CFTR KO mice (age 8-12 weeks, male), FVB/N background[3]) were kindly supplied by Dr. Ursula Seidler (Hannover Medical School, Hannover, Germany) and housed at the University of Szeged. Animals were kept in standard plastic cages on 12 hour light and dark cycle at room temperature and had free access to standard or CFTR specific laboratory chow and drinking solutions.[3] All mice were genotyped prior to the experiments. For genotyping, genomic DNA from the tail was isolated and amplified by traditional PCR. Ethical approval for the study was issued by the Public Health and Food Chain committee, Csongrad County Government Office, Hungary.

***In vivo* studies**

C57BL/6NHsd mice were age-matched and grouped based on their fasted blood glucose and body weight. The dosing regime is described in Figure S. In brief, animals were fasted for 4 hours prior to receiving once daily intraperitoneal (i.p.) injections of vehicle control (DMSO) or CFTRinh172 (3mg/kg body weight) for 8 consecutive days starting on Day -3. CFTRinh172 was administered alone or in combination with a low dose streptozotocin (STZ) regimen, which began on Day 1 of the study. Animals were fasted for 4 hours prior to receiving once daily i.p. injections of vehicle control (DMSO) or STZ (50 mg/kg body weight; made freshly in 0.1 M sodium citrate buffer, pH 4.5) for 5 consecutive days. Injections were administered at approximately 2PM each day. This resulted in the creation of 4 groups of animals: (1) vehicle control; (2) STZ-treated; (3) CFTRinh172-treated; and (4) STZ + CFTRinh172-treated. No adverse events were experienced during this study.

Food intake and body weight were monitored daily. Blood samples were collected from the cut tip of the tail vein of conscious mice at regular intervals throughout the study. Blood

glucose was monitored using a hand-held Ascencia Contour blood glucose meter (Bayer Healthcare, Newbury, Berkshire, UK). Glucose tolerance and insulin responses were evaluated on the final day of the study after i.p. injection of glucose (18 mmol/kg body weight) in overnight (15 hour) fasted mice.

After a 7-day washout period, mice were culled by Schedule 1 methods and pancreata, intestines and terminal blood were collected. Pancreata were halved longitudinally from head to tail with one half used for histology and the other half used for islet isolation or determination of islet hormone content as described below. Terminal analysis also included measurement of total body fat and lean mass by dual-energy X-ray absorptiometry (DEXA) scanning (Piximus Densitometer; Inside Outside Sales, Fitchburg, WI, USA).

**Measurements of insulin resistance and sensitivity**

To measure insulin resistance following ipGTT, HOMA-IR (Homeostasis model of insulin resistance) was calculated as follows: [fasting serum glucose x fasting serum insulin/22.5].[4] An assessment of beta cell function was performed using HOMA-B (Homeostasis model of beta cell function) as follows: [20* (fasting serum insulin/fasting serum glucose) – 3.5].[5] QUICKI (Quantitative insulin sensitivity check index) was also calculated for all animals as follows: [1/(log fasting serum glucose + log fasting serum insulin)].[6]

To further assess any evidence of insulin resistance in animals treated with STZ, CFTRinh172, or a combination of STZ + CFTRinh172, Western Blotting for phosphorylated IRS-1 was performed. Briefly, protein was extracted from the livers of animals using RIPA buffer (Thermo Scientific) supplemented with a protease inhibitor cocktail (Thermo Scientific). In brief, tissue was lysed in ice-cold RIPA buffer over 20 mins. A mechanical homogeniser was

used to aid lysis of the tissue. The lysate was centrifuged for 20 min at 14,000 rpm and 4 °C. The supernatant was transferred to a fresh eppendorf tube and the cellular protein content was assessed by BCA assay (ThermoFisher Scientific). Proteins (100 µg) were separated on a discontinuous SDS-PAGE NuPAGE Bis-Tris Electrophoresis System (ThermoFisher Scientific, UK) using pre-cast (4-12%) polyacrylamide mini-gels (Invitrogen, UK) and NuPAGE Running Buffer (ThermoFisher Scientific). Gels were run at 100 mA for 50 minutes. Separated proteins were transferred to PDVF membranes pre-soaked in methanol using NuPAGE Transfer Buffer at 170 mA for 60 minutes. The membranes were blocked overnight at 4 °C in 5% BSA dissolved in PBS, washed in PBS-T the following morning and incubated with primary antibody against phosphorylated IRS-1 (anti-Phospho-IRS1 Ser 612 (Cell Signalling #3193) overnight at 4 °C. Finally, the membranes were washed in PBS-T, incubated with secondary antibody (Anti-mouse IgG, HRP-linked Antibody (Cell Signalling #7076) at room temperature for 2 hours and washed for a final time with PBS-T. Membranes were exposed to SuperSignal West Pico Substrate (ThermoFisher) and imaged using Biolite UVP software (Biolite, US) and processed using Image J (nih.gov).

**Determination of islet hormone concentrations**

Commercially available ELISAs were used to measure insulin (Ultra-sensitive murine insulin ELISA, Mercodia), glucagon (R&D Systems) and GLP-1 (GLP-1 EIA kit, Sigma-Aldrich) concentrations according to the manufacturers' instructions. Plasma was isolated from tail vein and terminal blood samples and added directly to the ELISA plate. Islet hormone content was assessed following the extraction of protein from pancreatic tissue using RIPA buffer as described above for liver. A total of 5 µg protein was added to each well of the ELISA plates.

**Expression of islet regulatory genes**

The mRNA expression of islet regulatory genes was assessed by qPCR. mRNA was extracted from isolated mouse islets[7] using an RNeasy Mini Kit following manufacturer's instructions (Qiagen, UK). RNA was quantified in all instances using a NanoDrop (Thermo Scientific, UK). RNA quality was determined by a 260/280 ratio of 1.8 – 2.1 and through examination of RNA integrity on a 2% agarose gel (for a random selection of samples). mRNA (500 ng) was reverse transcribed to cDNA using transcriptor first strand cDNA synthesis kit (Roche, Burgess Hill, UK). UK). qPCR was performed on a Lightcycler 480 System (Roche, UK) using custom designed probes (Roche, see Table S1). Following optimization, B-actin was chosen as a reference gene for all experiments. 2 µl cDNA was used per 10 µl reaction and was mixed with 2 µl RNase-free water, 5 µl Master Mix (Roche Lightcycler, UK) and 1 µl relevant probe. All qPCR experiments including no RT controls, water controls and controls lacking probes. Each sample and control were added to a 96-well plate in duplicate and the plate was read according to an optimized protocol for monocolour hydrolysis probes provided by Roche, UK (annealing temperature of 60 °C). Relative mRNA expression was determined using $2^{\Delta Ct}$ method and normalised to beta-actin/ACTB expression.

**Immunofluorescent staining of pancreatic tissue**

Pancreatic tissues were immediately fixed in 4% PFA for 48 hours at 4 °C. The tissues were subsequently dehydrated using a series of increasing strength ethanol solutions and processed for embedding in paraffin wax using an automated tissue processor (Leica TP1020, Leica Microsystems, Nussloch, Germany), as described previously.[7] Tissue blocks were sectioned (4-5 µm) using a Shandon Finesse 325 microtome (Thermo Scientific, Hemel Hempstead, UK) and picked for staining at intervals of 10 sections. After dewaxing, sections were rehydrated using a series of decreasing strength ethanol solutions.[7] Antigen retrieval was carried out at

94 °C for 20 min using citrate buffer (pH 6.0). Sections were then blocked using 2% BSA or 5% goat serum and incubated overnight at 4 °C with appropriate primary antibody (Table 1). The slides were incubated with corresponding secondary antibodies (Table 1). Slides were mounted with anti-fade mounting medium with DAPI and viewed using a Nikon A1 confocal microscope (Nikon UK Limited, Surry, United Kingdom). All staining procedures and image analysis were carried out in a blinded manner.

**Assessment of IL-6 concentration**

Pancreatic IL-6 concentrations were measured following isolation of protein from pancreatic tissue as described in the ESM. IL-6 concentration was assessed using a commercially available murine ELISA kit (PeproTech EU). A total of 5 μg protein was added to each well of the ELISA plate and the assay was conducted in accordance with the manufacturer's instructions.

## RESULTS

### Short-term CFTR inhibition does not influence body composition of C57BL/6 mice

Treatment of animals with CFTRinh172 or a combination of STZ and CFTRinh172 did not impact bone mineral content, bone mineral density, lean mass or fat mass. However, STZ treatment negatively impacted fat mass and percentage fat (Figure S2).

### Short-term CFTR inhibition does not alter measures of insulin resistance or sensitivity in C57BL/6 mice

Treatment of animals with CFTR alone did not significantly alter the HOMA-IR, HOMA-B or QUICKI scores. Furthermore, there was no evidence of phosphorylated IRS-1 accumulation in the livers of these animals. In all instances, treatment with STZ was consistent with an insulin resistant phenotype (Figure S3).

### Short-term CFTR inhibition does not influence the mRNA expression of glucose-sensing genes

Islets were isolated from CFTR-inhibited animals and vehicle controls and the expression of several glucose-sensing genes examined. Short-term CFTR inhibition did not significantly influence the expression of *SCLC2A2, GCK, ABCC8* or *KCNJ11* (Figure S4).

### Pancreatic IL-6 concentrations are not influenced by short-term CFTR inhibition

The IL-6 content of pancreatic protein extract was determined by ELISA. None of the treatment conditions influenced IL-6 concentrations in the current study (Figure S5).

**Villus height is unchanged in mice treated with CFTRinh172 and in the ΔF508 mouse model.**

Intestinal villus height was not influenced by short-term CFTR inhibition consistent with the ΔF508 model (Figure S8)

**Short-term CFTR inhibition was insufficient to induce significant fibrosis in the pancreas or intestine**

Despite occasional evidence of additional collagen deposition around the pancreatic ducts and within the basement membrane of the intestine, the overall % area stained for Sirius red did not differ between control and CFTR-inhibited animals (Figure S9).

## TABLES

### Table S1: qPCR hydrolysis probe IDs (Roche)

| Target Gene | Assay ID |
|---|---|
| *SCLC2A2* | 311351 |
| *GCK* | 311106 |
| *ABCC8* | 300187 |
| *KCNJ11* | 317283 |
| *Nkx6.1* | 318473 |
| *Pdx-1* | 314873 |
| *Beta-actin* | 500152 |

### Table S2: Primary Antibodies

| Target | Host | Dilution | Source |
|---|---|---|---|
| Insulin | Guinea pig | 1:500 | Abcam (ab7842) |
| Glucagon | Mouse | 1:100 | Abcam (ab92517) |
| Somatostatin | Mouse | 1:500 | Abcam (ab30788) |
| Pancreatic polypeptide | Mouse | 1:200 | Abcam (ab77192) |
| Chromogranin A | Rabbit | 1:200 | Abcam (ab15160) |
| Nkx6.1 | Mouse | 1:100 | DSHB (F55A10) |
| Pdx-1 | Mouse | 1:200 | DSHB (F6A11-c) |

All antibodies were validated in control tissue and with negative controls lacking primary antibody.

### Table S3: Secondary Antibodies

| Target | Host | Reactivity | Dilution | Fluorescent dye and Source |
|---|---|---|---|---|
| IgG | Goat | Mouse | 1:1000 | Alexa Fluor® 594, Invitrogen (A-11032) |
| IgG | Goat | Guinea pig | 1:1000 | Alexa Fluor® 488, Abcam (ab150185) |
| IgG | Goat | Rabbit | 1:1000 | Alexa Fluor® 594, Abcam (ab150080) |

## FIGURES

**Figure S1: Treatment regimens for CFTR inhibition with or without hyperglycaemia.**

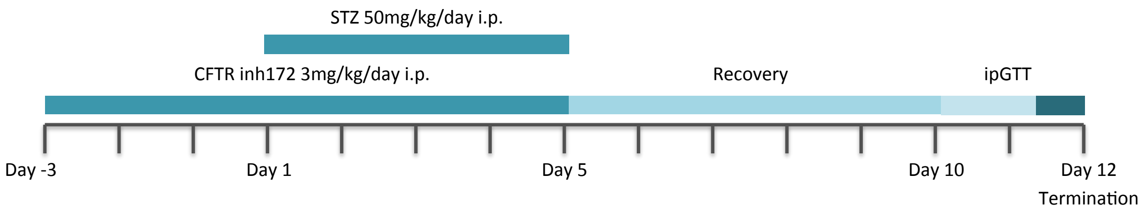


C57BL/6 mice were fasted for 4 hours prior to receiving once daily intraperitoneal (i.p.) injections of vehicle control (DMSO) or CFTRinh172 (3mg/kg body weight) for 8 consecutive days starting on Day -3. CFTRinh172 was administered alone or in combination with a low dose streptozotocin (STZ) regimen (50 mg/kg body weight/day for 5 consecutive days), which began on the Day 1 of the study. Animals were allowed to recover for a total of 7 days before being killed by schedule 1 methods. On the final day of the study, animals underwent ipGTT.

**Figure S2: Cumulative food and water intake and measurements of body composition.**

A.

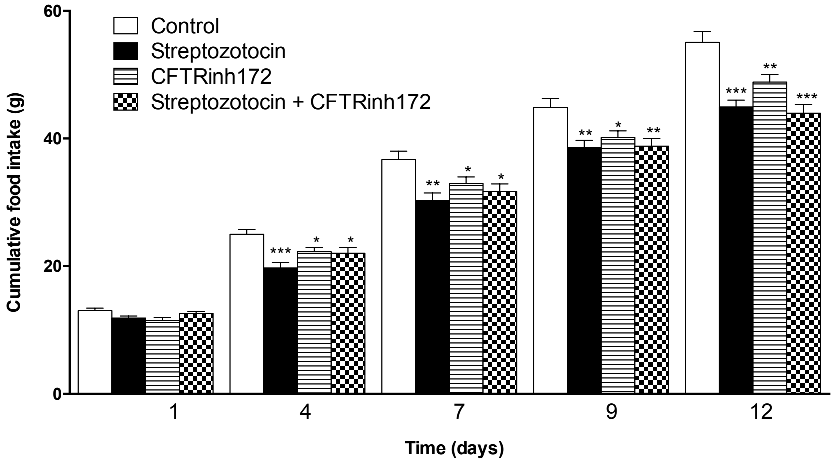


B.

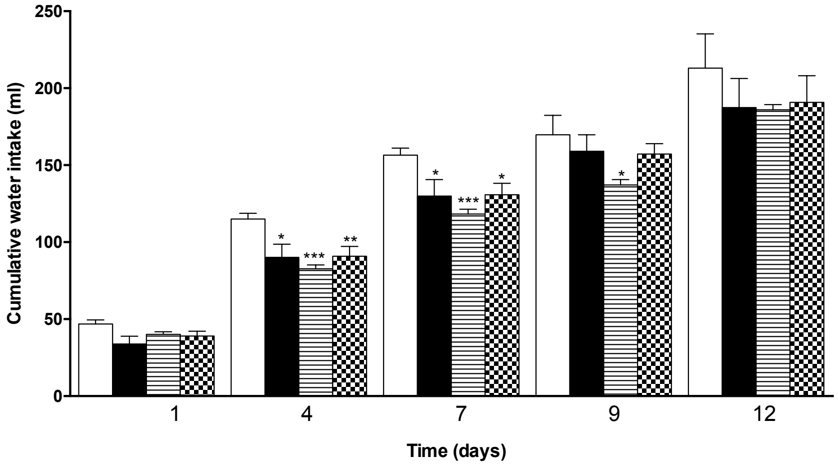


C.

| | Control | STZ | CFTRinh172 | STZ + CFTRinh172 |
|---|---|---|---|---|
| **BMD (g/cm²)** | 0.054 (0.0013) | 0.055 (0.0016) | 0.053 (0.0004) | 0.056 (0.0017) |
| **BMC (g)** | 0.499 (0.0189) | 0.545 (0.0300) | 0.492 (0.0115) | 0.566 (0.0283) |
| **Lean Mass (g)** | 22.68 (0.2366) | 21.66 (0.5425) | 22.83 (0.4573) | 22.4 (0.4091) |
| **Fat Mass (g)** | 3.675 (0.2491) | 2.738** (0.1782) | 3.28 (0.1241) | 2.3** (0.1483) |
| **% Fat** | 13.93 (0.8618) | 11.23* (0.3685) | 13.12 (0.753) | 9.36** (0.5618) |

(A) Cumulative food intake, (B) cumulative fluid intake, (C) Body composition of mice treated with vehicle control, STZ, CFTRinh172 and STZ + CFTRinh172. Values are mean ± SEM for n=7-8 mice. *$P$<0.05, **$P$<0.01 compared to healthy vehicle controls. BMD, bone mineral density; BMC, bone mineral content; STZ, Streptozotocin.

**Figure S3: Measures of insulin resistance and sensitivity**

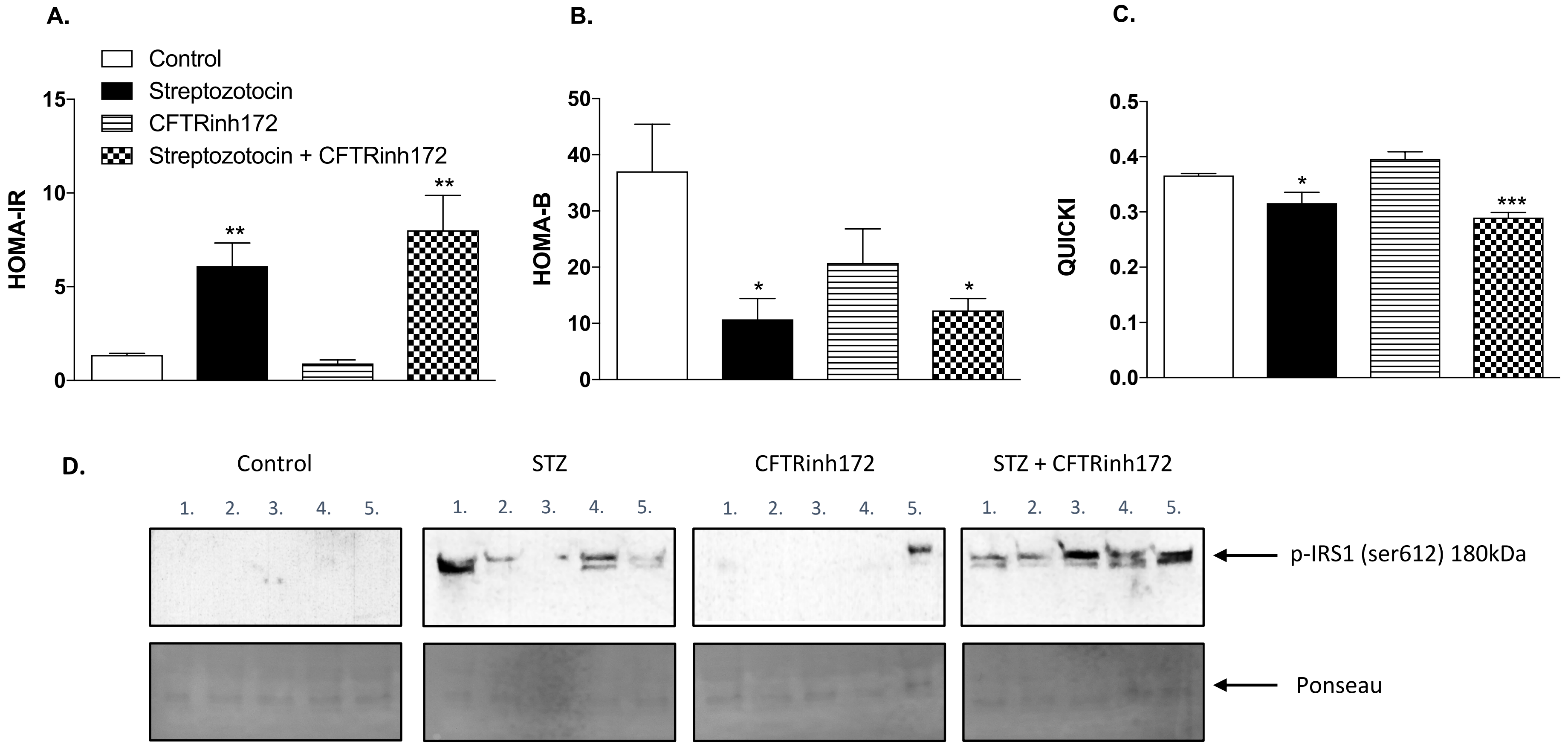


(A) HOMA-IR, (B) HOMA-B, (C) QUICKI scores for mice treated with vehicle control, STZ, CFTRinh172 and STZ + CFTRinh172. Values are mean ± SEM for n=6 mice. *$P$<0.05, **$P$<0.01, $P$<0.001 compared to healthy vehicle controls. (D) Western Blot analysis of phosphorylated IRS-1 and loading control (Ponseau) in liver extracts from mice treated with vehicle control, STZ, CFTRinh172 and STZ + CFTRinh172 (n=5 per group; each lane represents lysate from a different animal). STZ, Streptozotocin.

**Figure S4: mRNA expression of glucose-sensing genes**

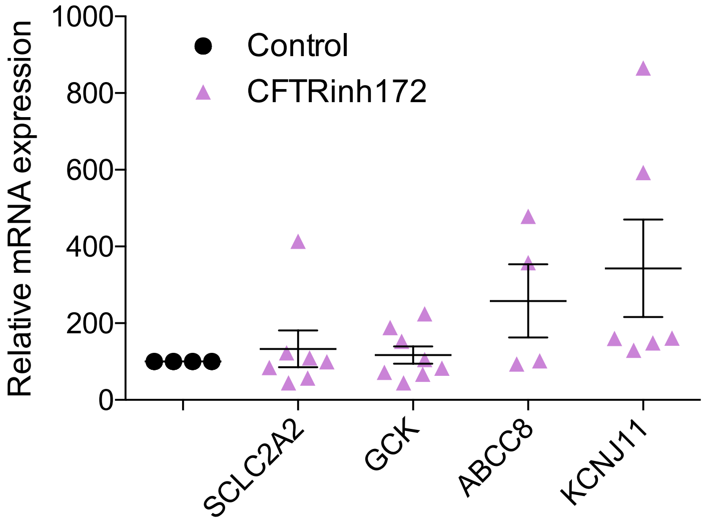


The effect of CFTR inhibition on the mRNA expression of glucose sensing genes in primary islets obtained from C57BL/6 mice was examined by qPCR. Data are expressed as a percentage of control. Results are presented as mean ± SEM (n=4-7).

**Figure S5: Pancreatic IL-6 concentrations**

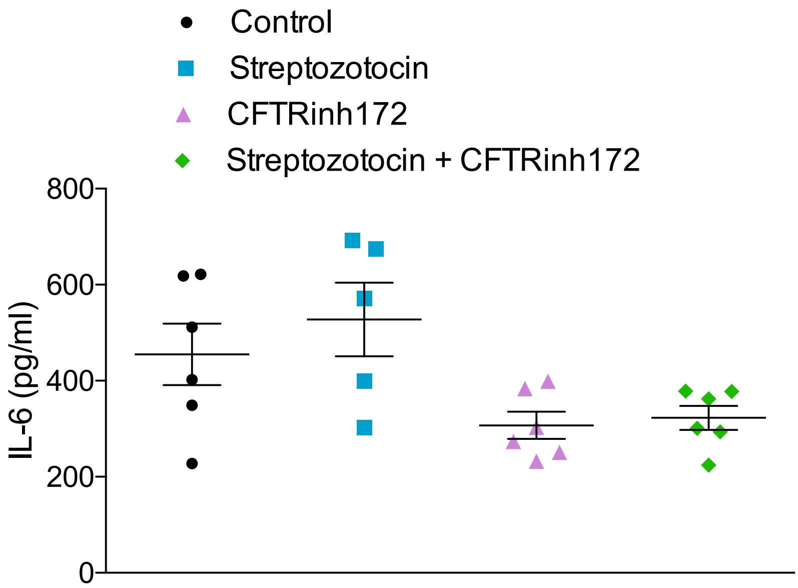


The effect of CFTR inhibition on pancreatic IL-6 concentration was examined by ELISA. Data are expressed as a percentage of control. Results are presented as mean ± SEM (n=6).

**Figure S6. H&E staining of the pancreas of transgenic ΔF508 mice**

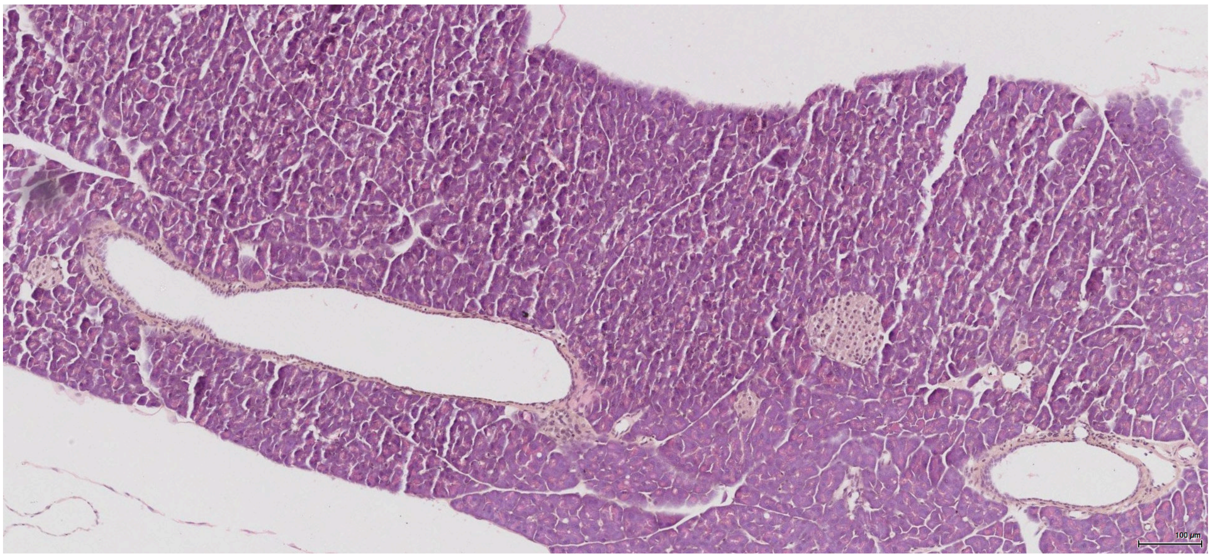

Shown above is the complete version of the composite image presented in the main text in Figure 8a.

**Figure S7. Higher-resolution images of the pancreas of mice treated with CFTRinh172**

Vehicle control CFTRinh172

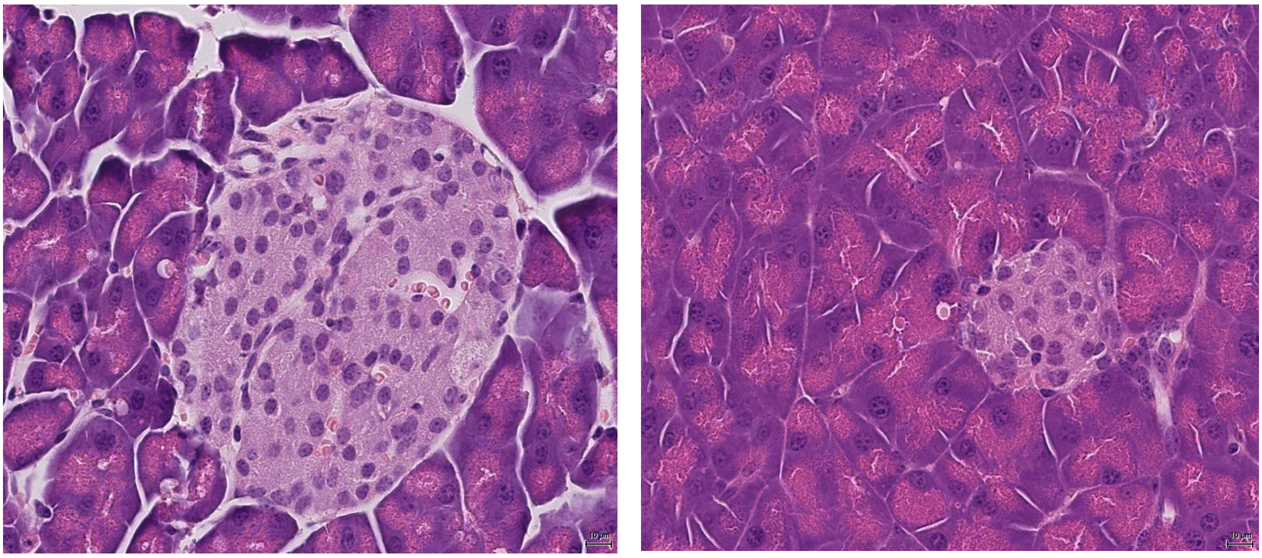

Presented above are representative higher resolution images (x40 magnification) of islets from mice treated with vehicle control or CFTRinh172. Fully searchable dynamic links to full pancreatic tissue sections from five individual mice are provided below:

Control animals:
http://nbb-slidepath.ncl.ac.uk/dih/webViewer.php?snapshotId=15439197192432

CFTRinh172-treated animals:
http://nbb-slidepath.ncl.ac.uk/dih/webViewer.php?snapshotId=15439193123393

**Figure S8: Villus height in CFTR-inhibited and ΔF508 mice**

a.

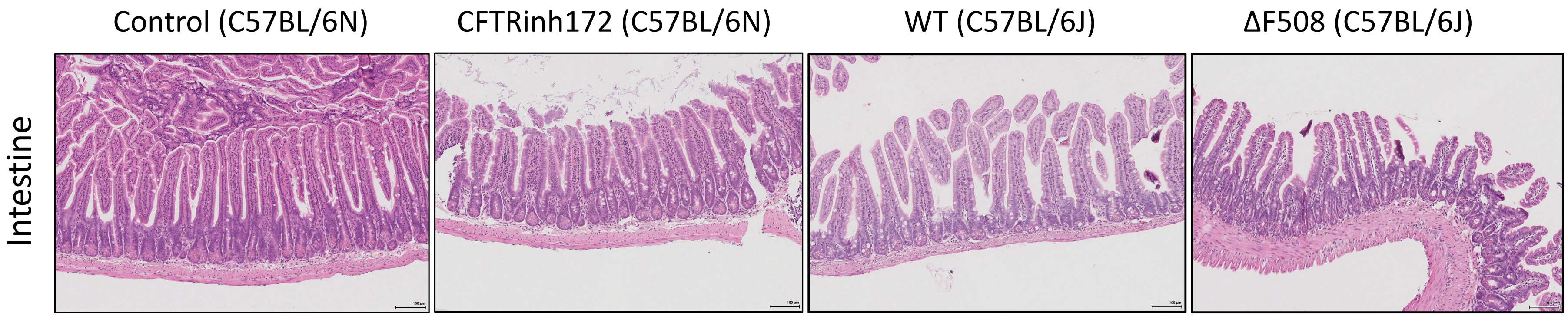


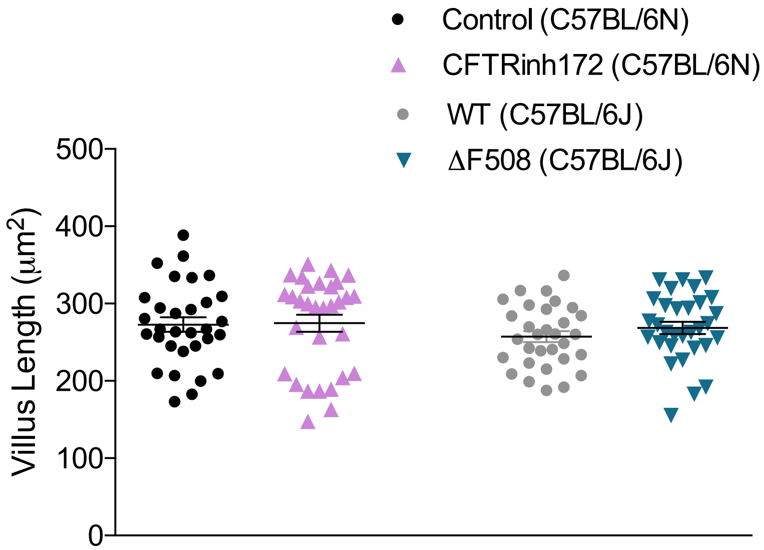


Representative H&E stained sections of the (a) intestine of CFTRinh172-treated mice and vehicle controls (both n=5) and transgenic ΔF508 mice and wild-type controls (both n=3). (b) intestinal villus length has been quantified. Values represent mean ± SEM with at least 10 data points collected per animal. Each data point on the graphs represents one villus.

**Figure S9. Fibrotic progression in the pancreas and intestine.**

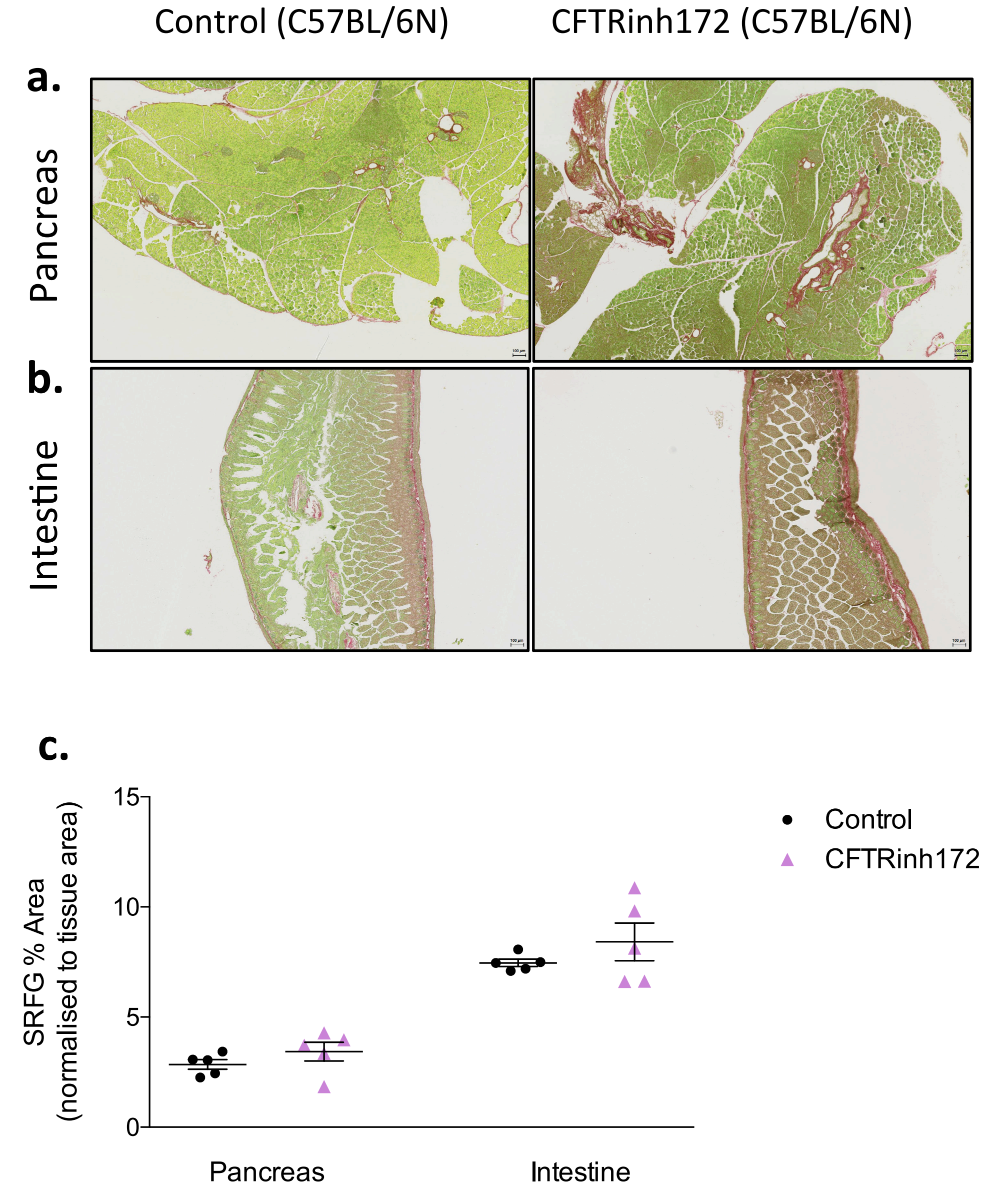


Representative sirius red/fast green (SRFG) stained sections of the (a) pancreas and (b) intestine of CFTRinh172-treated mice and vehicle controls (both n=5). (c) Collagen deposition has been calculated as a function of the SRFG % area and normalised to the entire tissue area. Values represent mean ± SEM.

## REFERENCES


1. van Doorninck, J. H., French, P. J., Verbeek, E., Peters, R., Morreau, H., *et al.* A mouse model for the cystic fibrosis delta F508 mutation. *EMBO J.* **14**, 4403-4411 (1995).
2. Svedin, E., Utorova, R., Hühn, M. H., Larsson, P. G., Stone, V. M., *et al.* A Link Between a Common Mutation in CFTR and Impaired Innate and Adaptive Viral Defense. *J. Infect. Dis.* **216**, 1308-1317 (2017).
3. Ratcliff, R., Evans, M. J., Cuthbert, A. W., MacVinish, L. J., Foster, D., Anderson, J. R., Colledge, W. H. Production of a severe cystic fibrosis mutation in mice by gene targeting. *Nat. Genet.* **4,** 35-41 (1993).
4. Fraulob, J. C., Ogg-Diamantino, R., Fernandes-Santos, C., Aguila, M. B., Mandarim-de-Lacerda, C. A. A Mouse Model of Metabolic Syndrome: Insulin Resistance, Fatty Liver and Non-Alcoholic Fatty Pancreas Disease (NAFPD) in C57BL/6 Mice Fed a High Fat Diet. *J. Clin. Biochem. Nutr.* **46**, 212–223 (2010).
5. Cersosimo, E., Solis-Herrera, C, Trautmann, M, E., Malloy, J., Triplitt, C. L. Assessment of Pancreatic β-Cell Function: Review of Methods and Clinical Applications. *Curr. Diabetes Rev.* **10,** 2–42 (2014).
6. Mather, K. Surrogate measures of insulin resistance: of rats, mice, and men. *Am. J. Physiol. Endocrinol. Metab.* **296,** E398–E399 (2009).
7. Khan, D., Vasu, S., Moffett, R. C., Irwin, N., Flatt, P. R. Islet distribution of peptide YY and its regulatory role in primary mouse islets and immortalised rodent and human beta-cell function and survival. *Mol. Cell. Endocrinol.* **436,** 102-113 (2016).
